# Comparison of the montreal cognitive assessment and the mini-mental state examination as screening tests in hemodialysis patients without symptoms

**DOI:** 10.1080/0886022X.2018.1455589

**Published:** 2018-04-10

**Authors:** Sun Hwa Lee, AJin Cho, Yang-Ki Min, Young-Ki Lee, San Jung

**Affiliations:** a Department of Neurology, Hallym University Medical Center Kangnam Sacred Heart Hospital, Seoul, Korea;; b Department of Neurology, Hallym University Medical Center Dongtan Sacred Heart Hospital, Seoul, Korea;; c Department of Internal Medicine, Division of Nephrology, Hallym University Medical Center Kangnam Sacred Heart Hospital, Seoul, Korea

**Keywords:** Cognitive impairment, hemodialysis patients, chronic kidney disease, MoCA

## Abstract

Cognitive impairment in end-stage renal disease patients is associated with an increased risk of mortality. We examined the cognitive function in hemodialysis (HD) patients and compared the Korean versions of the Montreal Cognitive Assessment (K-MoCA) and of the Mini-Mental State Examination (K-MMSE) to identify the better cognitive screening instrument in these patients. Thirty patients undergoing hemodialysis and 30 matched reference group of apparently healthy control were included. All subjects underwent the K-MoCA, K-MMSE and a neuropsychological test battery to measure attention, visuospatial function, language, memory and executive function. All cognitive data were converted to z-scores with appropriate age and education level prior to group comparisons. Cognitive performance 1.0 SD below the mean was defined as modest cognitve impairment while 1.5 below the mean was defined as severe cognitive impairment. Modest cognitive impairment in memory plus other cognitive domains was detected in 27 patients (90%) while severe cognitive impairment in memory plus other cognitive domains was detected in 23 (77%) patients. Total scores in the K-MoCA were significantly lower in HD patients than in the reference group. However, no significant group difference was found in the K-MMSE. The K-MMSE ROC AUC (95% confidence interval) was 0.72 (0.59–0.85) and K-MoCA ROC AUC was 0.77 (0.65–0.89). Cognitive impairment is common but under-diagnosed in this population. The K-MoCA seems to be more sensitive than the K-MMSE in HD patients.

## Introduction

End-stage renal disease (ESRD) has an impact on quality of life, potentially affecting patients’ physical, mental health, and functional status. Since the 1980s changes in cognitive function are a well-known consequence of ESRD [1] and may become an important public health issue in hemodialysis (HD) patients. The etiology of cognitive impairment in HD patients is thought to be multifactorial. The high prevalence of cardiovascular risk factors, cerebrovascular lesions, hemodynamic instability during dialysis, serological abnormalities (uremia, anemia, metabolic disturbances), measures of malnutrition and hemodialysis volume have been reported as contributing factors [2–7].

The MMSE [8] is the most widely used cognitive screening instrument worldwide, available in a multitude of translations, and validated in many clinical populations. However, the instrument has proven to be relatively insensitive to conditions associated with frontal-executive and to milder forms of cognitive impairment [9]. Previous studies using more than two cognitive tests revealed that HD patients were more than 3 times as likely to have severe cognitive impairment than non-HD patients while only 2.9% of the patients had a documented history of cognitive impairment [6,10]. The presence of cognitive impairment seems relatively high when more than two cognitive tests were used however, subjective cognitive complain seems to be infrequent in this population. It is difficult to motivate patients to undergo more than two cognitive tests when they have no subjective complain. Therefore, it seems necessary to adopt a more qualitatively detailed but relatively simple screening test for this population.

The Montreal Cognitive Assessment (MoCA) was developed to screen patients with mild cognitive complaints and showed higher sensitivity [11]. Compared to the MMSE, the MoCA contains a greater variety of subtests making it a potentially more sensitive battery to assess diverse cognitive domains such as attention, executive and visuospatial functions.

Recently, the MoCA was recommended as a screening test for detecting cognitive impairment in HD patients [12]. However, few studies have assessed the MoCA in this population particularly using normative data with appropriate age and education level. In the present study, we examined the cognitive function in HD patients and compared two commonly used screening tools, the K-MMSE and the K-MoCA to identify cognitive deficits. All cognitive test scores including screening tests were analyzed using normative data stratified by age and education in a large, population-based sample.

## Methods

### Participants and procedures

Thirty patients, who were undergoing hemodialysis in the Department of Nephrology of the Hallym University Kangnam Sacred Heart Hospital, were recruited in this study as HD group. Patients were asked to participate if they met the following inclusion criteria: (1) aged over 50 years or more, (2) no clinical history of neurological disorders such as stroke or dementia, (3) no clinical history of psychiatric disorders such as depressive disorder or anxiety disorder. No one had hypothyroidism. Of 34 approached patients 30 agreed to participate in this study. Thirty matching reference group in terms of age, education level and sex ratio was recruited amongst the *informants* of outpatients in the Department of Neurology of the same institute. Self-report of good health with normal renal function, confirmed by laboratory data from the primary care physician within 12 months of testing, no clinical history of neurological or psychiatric diseases, and currently not taking any medication were the inclusion criteria for the reference group and considered as *apparently healthy*. All participants were asked two questions: (1) Are you currently experiencing cognitive decline and if so (2) does it cause any difficulties in your activities of daily living (ADL). A negative answer to both was considered as no subjective symptoms present and normal ADL. Written informed consent was obtained from all participants prior to study participation. The study protocol and analysis plan were approved by the research ethics board at the Hallym University Kangnam Sacred Heart Hospital. The procedures followed were in accordance with the ethical standards of the responsible committee on human experimentation both institutional and national and with the Helsinki Declaration of 1975.

A trained clinical neuropsychologist [S.H.L] administered the K-MoCA, K-MMSE, and the comprehensive neuropsychological test battery on one day at the neurocognitive laboratory of the Department of Neurology. All participants underwent comprehensive neuropsychological test battery in the same order. The K-MMSE and K-MoCA were administered either before or after the detailed cognitive battery and the sequence was counterbalanced. The average time requirement for the overall test was 1.5 h. HD patients were administered the test on an appointed dialysis day at least 1 or 2 h prior to the HD therapy.

### Assessments

Demographic, clinical data and medication details were collected from patients’ medical records. The routine clinical chemistry and complete blood count results were recorded on the day of cognitive assessment for HD group.

The neuropsychological test battery included measures in the following 5 cognitive domains: (1) attention (digit span forward [DSF] and digit span backward [DSB]), (2) visuospatial function (Rey Complex Figure Test copying task [RCFT]), (3) language (Short form of the Korean-Boston Naming Test [S-K-BNT]: 15 items), (4) memory (Seoul Verbal Learning Test: 3 trials of a 12-word list learning [SVLT-IR], 20-min delayed free recall [SVLT-DR], and recognition [SVLT-R]), and (5) executive function and working memory (verbal fluency test; semantic [VF-S] and phonemic fluency [VF-P] tests, Korean version of Stroop Color-Word Test [K-SCWT] color reading task, Digit Symbol Coding Test [DSCT], and Trail Making Test part A [TMT-A] and part B [TMT-B]. All tests were adopted from the Seoul Neuropsychological Screening Battery (SNSB) and raw scores were transformed to standardized z-scores using the published normative data with appropriate age and education level [13]. The mean and standard deviation was adopted from the SNSB normative data of 1067 subjects. The raw score, total numbers of correct, for DSF, DSB, RCFT, S-K-BNT, SVLT, VF-S, VF-P and DSCT, was subtracted by the mean score then divided by the standard deviation. The raw score, the time (sec) required to complete the test, for TMT-A and TMT-B, was subtracted by the mean score then divided by the standard deviation. For example, if a HD patient who are a male, aged 66 with education level of 9 years, performed total correct of 4 in DSF test, the mean was 5.76 with standard deviation of 1.27 in the normative data aged between 65–69 with education level between 7–9 years. The standard z-score is −1.39. The Korean version of the Geriatric Depression Scale [GDS], which contains 30 yes or no questions with a cutoff value of 18, was also administered to assess depression [14].

The MoCA is a brief screening instrument composed of 12 items assessing visuospatial and executive function (5 points), naming (3 points), attention (6 points), abstraction (2 points), short term memory (5 points) and orientation (6 points) sequentially. The maximum score is 30 and higher scores indicate higher cognitive function. There are two versions, the MoCA-K and K-MoCA, available in Korean language with slight differences in test items relative to cultural/language characteristics while maintaining the basic framework of the original MoCA. The K-MoCA, which is validated more recently with normative data, stratified by age and education level is available, and was adopted for this study [15].

The MMSE is the most commonly used screening test including items assessing orientation (5 points for temporal orientation, 5 points for spatial orientation), memory (3 points for immediate recall, 3 points for delayed recall), serial subtraction (5 points), language ability (2 points for naming, 3 points for oral command comprehension, 1 point each for repetition, reading, and for writing), and visuospatial ability (1 point) in order. The maximum score is also 30 where higher scores indicate higher cognitive function. Two versions of MMSE in Korean are available; the K-MMSE, which has been validated more recently with normative data stratified by age and education level, is available and was adopted in this study.

### Data analysis

The normal distribution was tested using the Kolmogorov–Smirnov test. Test results are reported as mean and standard deviation (SD) for normally distributed continuous variables, while median and interquartile range were presented for non-normally distributed continuous variables. Demographic and clinical data are presented according to the respective distribution type. Mann–Whitney U-test with Bonferroni correction was used for the nonparametric data while independent-sample *t*-test with *Levene’s* test for equality of variances was used for parametric data. The effect size *r* for all variables was calculated according to Cohen’s proposal where *r* < 0.2 is a small effect, a *r* between 0.30 and 0.5 is a moderate effect, and *r* > 0.5 indicates a large effect [16]. Pearson’s chi-square test was performed for categorical variables. Correlational analysis was performed using Spearman’s’ rank correlation coefficient (*r_s_*).

Raw scores from the neuropsychological tests and the K-MMSE total score were transformed to z-scores based on normative data from the Seoul Neuropsychological Screening Battery 2nd edition [13]. Normative data was obtained from 1067 (male: 600, female 467) healthy controls and was stratified by 9 age groups (45–49, 50–54, 55–59, 60–64, 65–69, 70–74, 75–79, 80–84, 85–90 years old) and 7 education levels (illiterate group, literate to 3 years, 4–6, 7–9, 10–12, 13–16, more than 17 years education). The K-MoCA total score was standardized with a z-score based on normative data from the validation conducted by Kang and colleagues [15] obtained from 398 (male: 173, female: 225) healthy elderly subjects stratified by 6 age groups (50–64, 55–69, 60–74, 65–79, 70–84, 75–90 years old) and 5 education levels (illiterate group, literate to 3 year, 4–6, 7–12, more than 13 years education). The RCFT z-score was used as the visuospatial domain score and the z-score of S-K-BNT was used as the language domain score. We calculated composite scores for attention (DSF and DSB), memory (SVLT-IR, SVLT-DR, and SVLT-R), and executive functions (VF-S, VF-P, K-SCWT, DSCT, and TMT-B) where multiple tests were used. The composite score was calculated by averaging the z-scores of each subtest.

Cognitive impairment was defined by both 1.0 SD and 1.5 SD below the published normative data. Cognitive performance 1.0 SD below the mean was defined as modest cognitive impairment while 1.5 below the mean was defined as severe cognitive impairment. A bivariate linear regression model was also calculated using the z-score of the K-MMSE or K-MoCA as the independent variable and the total cognitive composite score as the dependent variable.

Receiver operating characteristic (ROC) curves with area under the curve (AUC) (95% confidence interval [95% CI]) was plotted to compare the appropriateness of the K-MMSE or the K-MoCA to differentiate the cognitive impairment from normal cognition.

## Results

Demographic characteristics of the participants are reported in Table 1. There were no significant group differences in age, sex ratio, education or depression level between patient and reference group. None of the participants complained of subjective cognitive impairment or impairment in ADL. Clinical history and laboratory test results were also demonstrated in Table 1.

**Table 1.** Demographic characteristics of the study participants.

Variable	HD group (*n* = 30)	Reference group (*n* = 30)	*p* value
Age (years)	64.90 ± 7.88	68.40 ± 6.35	.063
Sex, male, *n* (%)	12 (40)	9 (30)	.417
Education (years)	9.57 ± 3.74	9.80 ± 3.58	.806
GDS (cutoff ≥18)	14.60 ± 7.09	13.38 ± 3.93	.477
Hypertension, *n* (%)	22 (73.33)	0	<.001
Diabetes, *n* (%)	12 (40)	0	<.001
Hemoglobin (g/dL)	10.42 ± 0.99		
Albumin (g/dL)	4.04 ± 0.44		
CRP (mg/L)	5.00 ± 7.98		
HDL-C (mg/dL)	40.35 ± 10.49		
intact PTH (pg/mL)	189.25 ± 164.53		
Duration of hemodialysis (months)	52.08 ± 50.89		
ACEi or ARB, *n* (%)	0.85 ± 0.37		
Beta-blockers, *n* (%)	0.58 ± 0.50		
Calcium channel blockers, *n* (%)	0.58 ± 0.50		

ACEi: angiotensin-converting-enzyme inhibitor; ARB: angiotensin-receptor-blocker; CRP: C-reactive protein; GDS: Geriatric Depression Scale; HDL-C: high-density lipoprotein cholesterol; PTH: parathyroid hormone.

Individual neuropsychological tests scores were converted to z-scores and group differences were analyzed. HD patients performed significantly worse than reference group in all cognitive domains including all of the neuropsychological subtests (Table 2). The performance on the TMT-B was the poorest in the patients group followed by the RCFT, SVLT-DR, TMT-A, and K-SCWT as worst 5 in sequential order.

**Table 2.** Standard z-score for 5 cognitive domains and 13 cognitive measures.

	HD group (*n* = 30)	Reference group (*n* = 30)	*p* value	Effect size *r*
Attention	−0.78 ± 0.64	0.41 ± 0.97	<.001	0.59
DSF	−0.73 ± 0.68	0.18 ± 0.84	<.001	0.51
DSB	−0.55 ± 0.78	0.56 ± 1.25	<.001	0.47
Visuospatial				
RCFT	−1.42 ± 1.95	0.08 ± 0.55	<.001	0.46
Language				
S-K-BNT	−1.07 ± 0.57	0.68 ± 0.87	<.001	0.61
Memory	−1.47 ± 1.19	0.54 ± 0.46	<.001	0.74
SVLT-IR	−0.77 ± 0.79	0.44 ± 0.75	<.001	0.62
SVLT-DR	−1.36 ± 0.86	0.19 ± 0.52	<.001	0.74
SVLT-R	−0.83 ± 1.28	0.54 ± 0.71	<.001	0.55
Executive function	−1.72 ± 3.05	0.86 ± 0.69	<.001	0.50
VF-S	−0.80 ± 0.83	0.27 ± 0.99	<.001	0.53
VF-P	−1.07 ± 0.57	0.68 ± 0.87	<.001	0.77
K-SCWT	−1.08 ± 1.12	0.33 ± 0.82	<.001	0.58
DSCT	−0.68 ± 1.16	0.88 ± 0.84	<.001	0.61
TMT-A	−1.29 ± 3.26	0.62 ± 0.66	<.004	0.38
TMT-B	−1.73 ± 2.96	0.52 ± 0.44	<.001	0.47

Values are presented as mean ± SD for normally distributed continuous variables, median (interquartile range) for non-normally distributed continuous variables. (1) attention (DSF, digit span forward and DSB, digit span backward), (2) visuospatial function (RCFT, Rey Complex Figure Test copying), (3) language (S-K-BNT, Short form of the Korean-Boston Naming Test: 15 items), (4) memory (SVLT-IR, Seoul Verbal Learning Test: 3 trials of a 12-word list learning, SVLT-DR, 20-min delayed free recall, and recognition [SVLT-R]), and (5) executive function and working memory (verbal fluency test; VF-S, semantic and VF-P, phonemic fluency tests; K-SCWT, Korean version of Stroop Color-Word Test; DSCT, Digit Symbol Coding Test, TMT-A, Trail Making Test part A; TMT-B, Trail Making Test part B).

The number of patients who showed 1.0 SD and 1.5 SD below the mean was displayed in Table 3. Memory deficit was the most frequent, executive dysfunction was second frequent. Of modest cognitive impairment, 18 (60%) HD patients demonstrated memory impairment, 13 (43%) had executive dysfunction and 27 (90%) showed impairment in memory plus one or more cognitive domain. Of severe cognitive impairment, 16 (53%) had memory impairment, 12 (40%) had executive dysfunction and 23 (77%) had impairment in memory plus other cognitive domain. When composite score, the average of 5 cognitive domain z-scores, was used, 15 (25%) of HD patients were modest cognitive impairment and 9 (30%) were severe cognitive impairment.

**Table 3.** Numbers of cognitive domain impaired with modest cognitive impairment (1.0 SD below the mean) and severe cognitive impairment (1.5 SD below the mean) in HD group (*n* = 30).

	Modest cognitive impairment (1.0 SD below the mean)	Severe cognitive impairment (1.5 SD below the mean)
Attention	12 (40%)	5 (17%)
Visuospatial	15 (50%)	7 (23%)
Language	12 (40%)	7 (23%)
Memory	18 (60%)	16 (53%)
Executive function	13 (43%)	12 (40%)
Memory plus other cognitive domain	27 (90%)	23 (77%)
Composite score	15 (50%)	9 (30%)

There was no significant group difference in K-MMSE scores regardless of whether the total score or the standardized z-score was used (Table 4). However, HD patients achieved lower scores in the K-MoCA and significant group differences were found in the total score and the standardized z-score. K-MoCA was significant at 2.5% using the total score, while it was significant at 0.2% when standardizing the total score to the z-score.

**Table 4.** The total scores and the z-scores of the K-MMSE and the K-MoCA.

	HD group (*n* = 30)	Reference group (*n* = 30)	*p* value	Effect size *r*
K-MMSE total score	26.35 ± 2.77	27.36 ± 1.47	.112	0.22
K-MMSE z-score	−0.98 ± 1.53	−0.27 ± 0.86	.052	0.28
K-MoCA total score	20.35 ± 4.54	22.92 ± 3.11	.025	0.31
K-MoCA z-score	−0.27 ± 1.27	0.34 ± 0.93	.002	0.26

**Figure 1.**
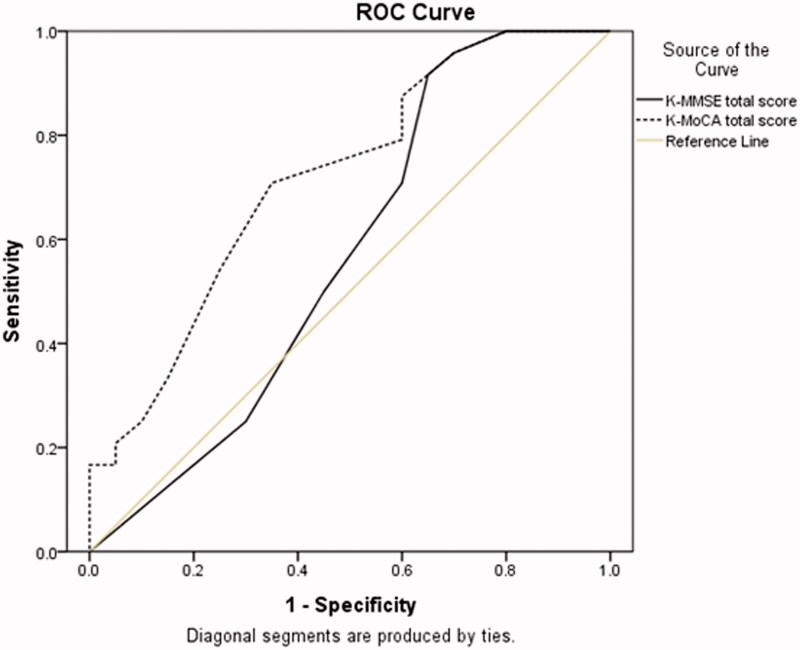
Receiver operating characteristic curve analysis: K-MMSE versus K-MoCA using total scores/raw score.

**Figure 2.**
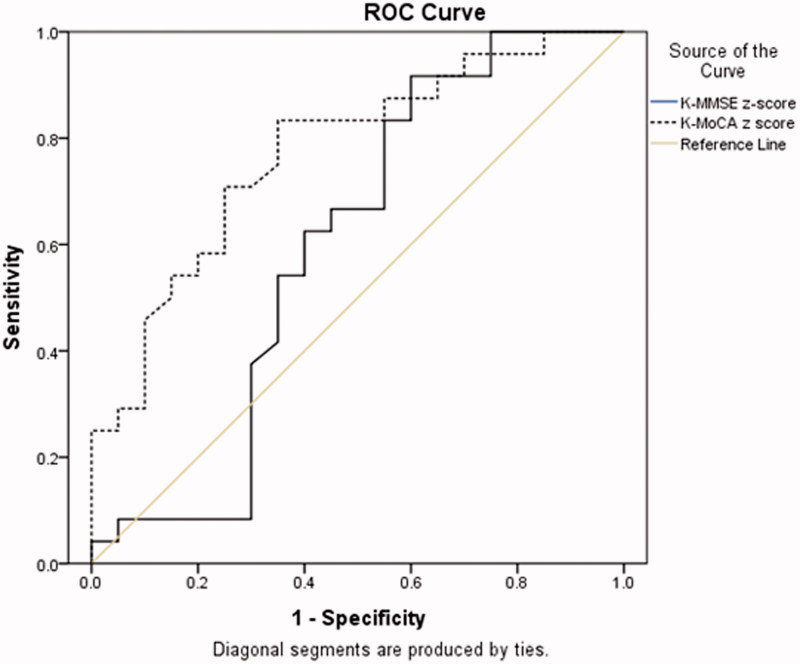
Receiver operating characteristic curve analysis: K-MMSE versus K-MoCA using z-scores.

ROC curves were drawn to compare the discrimination ability of the K-MMSE and the K-MoCA (Figures 1 and 2). The 27 modest cognitive impairment in memory plus other cognitive domain of the patient group were set as the 1 specificity. The ROC AUC, when using the total score, was higher in the K-MoCA (0.77, 95% CI 0.65–0.89) compared to K-MMSE (0.72, 95% CI 0.59–0.85). Similar finding were revealed when the total scores were converted to age- and education-adjusted z-score between the K-MMSE (0.75, 95% CI 0.62–0.88) versus the K-MoCA (0.77, 95% CI 0.63–0.91). These findings suggest K-MMSE did not differentiate cognitive impairment in HD patients who were literate and in patients >50 years old regardless of standardizing the total score by age or education level, while the K-MoCA does.

The Spearman’s rank correlation analysis was conducted using the z-scores for each variable. There was a small positive correlation between the K-MMSE and the K-MoCA (*r_s_* = 0.33, *p* = .005). A strong positive correlation between the total cognitive composite score and the K-MoCA was found (*r_s_* = 0.67, *p* < .001). The K-MoCA had moderate positive correlations with the executive (*r_s_* = 0.56, *p* < .001), the language (*r_s_* = 0.57, *p* < .001), the memory (*r_s_* = 0.42, *p* < .001), and the attention (*r_s_* = 0.42, *p* < .001) domains while there was a small positive relationship with the visuospatial (*r_s_* = 0.37, *p* = 0.002) domain. Linear regression analysis revealed 66.1% (β = 0.94, *p* < .001) of the variance in the K-MoCA was explained by the total cognitive composite score. However, the K-MMSE explained only 47.3% (β = 0.65, *p* < .001) of the variance in the total cognitive composite score.

## Discussion

This study showed that total scores of K-MoCA were significantly lower in HD patients than in a reference group. However, K-MMSE total scores were not significantly different between the two groups. K-MoCA was more sensitive than K-MMSE in HD patients. Early studies reported moderate rates of cognitive impairment across multiple cognitive domains in HD patients [6,10]. As in previous studies, cognitive impairment was also found in our data. Despite none of the patients complaining of subjective cognitive impairment and reporting no difficulties in ADL, all HD patients showed impaired performance in at least one of the cognitive domains; 90% had modest cognitive impairment in memory or memory with other cognitive domains and 77% had severe cognitive impairment in memory or memory with other cognitive domains. This implies that even without subjective cognitive impairment, modest to severe levels of cognitive impairment might be common and undiagnosed in HD patients. The memory domain was the most frequently impaired and there appeared to be particular difficulties in retrieval. The patients also showed poor performance in the naming test, but performance improved when a semantic or phonemic cue was presented. This could be interpreted as poor naming ability caused by retrieval failure. Retrieval of verbal information was found to be related to prefrontal areas in a functional brain imaging study [17]. Poor performance on the VF-P, the K-SCWT and the DSCT suggested that deficits in the executive function were also prominent in the patients group. Patients with kidney disease seemed to have particular difficulties in frontal lobe or executive dysfunction that may have partly resulted in anosognosia of their cognitive dysfunction. To check the patterns of cognitive deficit in specific domains may help the clinician’s treatment plan in terms of conversion to dementia and types of dementia likely to be encountered.

Several studies have revealed the effect of uremic toxins on neurons attributing this to the impairment of cognitive function in this population [18,19]. However, the persistence of cognitive impairment despite clinically adequate dialysis indicates that other factors might also contribute to brain dysfunction. Stroke and the high prevalence of cardiovascular risk factors have been powerful risk factors for the development of cognitive impairment [19,20]. ESRD might surrogate for accelerated atherosclerosis. In accordance with these studies, frontal lobe and executive dysfunction, which is correlated to the frontal lobe dysfunctions often observed in cerebrovascular disease, were found to be the most impaired cognitive domains in our data. Anemia and serum albumin in patients with ESRD have been associated with cognitive impairment [21,22]. Animal studies have focused on the potential role of secondary hyperparathyroidism as a risk factor for cognitive impairment in the CKD population [23,24]. In this study, C-reactive protein level and serum albumin were not correlated with K-MoCA or MMSE scores. Meanwhile hemoglobin levels significantly correlated with K-MoCA z-scores (*r_s_* = 0.36, *p* = 0.002). Parathyroid hormone level was positively correlated with K-MoCA (*r_s_* = 0.5, *p* = 0.003) and MMSE (*r_s_* = 0.45, *p* = 0.005) z-scores.

In this study, the K-MoCA demonstrated high sensitivity for screening patients with cognitive impairment. However, there were no significant differences in the K-MMSE score between HD patients and the controls. Most people receive lower scores on the MoCA than the MMSE when the MoCA and MMSE are combined. Although not compared individually, previous studies commonly report that the average MoCA score is lower than that of the MMSE [11,25]. This discrepancy, however, is likely related to the differences in item difficulty, rather than in a reduced MoCA sensitivity in identifying cognitive dysfunction. The difference in item difficulty of MMSE and MoCA is understandably different since the MoCA does not score any points for word registration while MMSE gives 3 points for three-words registration [26]. Such differences in scores were shown in normal healthy elderly subjects and suggest that MoCA could be a difficult test compared to the MMSE. Items with a high level of difficulty included in MoCA could be useful for detecting cognitive dysfunction at earlier stages of disease progression.

To minimize confounding variables potentially affecting cognitive function, all cognitive data were converted to standardized z-scores from age- and education-matched published normative data prior to group comparison. The primary advantage of utilizing a normative approach versus a cutoff score approach is that means and SDs may be stratified by different factors allowing for a more accurate estimate of cognitive performance. Although cutoff scores may provide good indications of sensitivity and specificity for differentiating clinical groups from controls, they are unable to account for significant confounding factors that may increase the likelihood of misclassification.

There are several limitations in this study. The small sample size might have low power for generalizability of our findings. Although effect sizes of many important factors in our data were moderate to large, further studies with larger sample sizes may confirm these findings. In addition, clinical data such as cardiovascular risk factors for the reference group were limited. Brain imaging data would enrich our knowledge about patients in HD treatment and suggest proper management by confirming possible asymptomatic strokes, such as silent infarcts, white matter disease, or leukoaraiosis. As Murray et al suggested that the best time to assess the cognitive abilities is on the day after dialysis or immediately before the dialysis session, we tested the HD patients 1–2 h before the dialysis session. However, Schineider and colleagues [27] demonstrated improvements in psychomotor speed, memory, and executive functions after a single dialysis session and recommended to test on non-dialysis day [28]. To test cognitive abilities immediately before the dialysis might be another possible bias and the results should be interpreted carefully. Moreover, the reference group of our study did not undergo the same medical screening and diagnostic examination as done for the HD group. Future study may test the same laboratory test and adopt more specific standard for CKD such as eGFR [29].

In conclusion, the MoCA is a valid screening test for cognitive impairment in HD patients. It demonstrated higher sensitivity for screening patients with mild cognitive impairment than MMSE. Cognitive impairment is often under-diagnosed due to the patients’ unawareness of their cognitive deficits, and thus, may make comprehensive neuropsychological examination more difficult in patients who do not subjectively experience cognitive deficits. Therefore, screening tests would be more appropriate for these patients. The K-MoCA, which is sensitive to executive dysfunction, seems to be more adaptable to HD patients.
